# Modeling *In Vitro* Cellular Responses to Silver Nanoparticles

**DOI:** 10.1155/2014/852890

**Published:** 2014-10-09

**Authors:** Dwaipayan Mukherjee, Steven G. Royce, Srijata Sarkar, Andrew Thorley, Stephan Schwander, Mary P. Ryan, Alexandra E. Porter, Kian Fan Chung, Teresa D. Tetley, Junfeng Zhang, Panos G. Georgopoulos

**Affiliations:** ^1^Environmental and Occupational Health Sciences Institute (EOHSI), Rutgers University, Piscataway, NJ, USA; ^2^Department of Environmental and Occupational Medicine, Robert Wood Johnson Medical School, Rutgers University, Piscataway, NJ, USA; ^3^Department of Chemical and Biochemical Engineering, Rutgers University, Piscataway, NJ, USA; ^4^Department of Environmental and Occupational Health, School of Public Health, Rutgers University, Piscataway, NJ, USA; ^5^National Heart and Lung Institute, Imperial College London, London, UK; ^6^Department of Materials and London Centre of Nanotechnology, Imperial College London, London, UK; ^7^Nicholas School of Environment and Duke Global Health Institute, Duke University, Durham, NC, USA

## Abstract

Engineered nanoparticles (NPs) have been widely demonstrated to induce toxic effects to various cell types. *In vitro* cell exposure systems have high potential for reliable, high throughput screening of nanoparticle toxicity, allowing focusing on particular pathways while excluding unwanted effects due to other cells or tissue dosimetry. The work presented here involves a detailed biologically based computational model of cellular interactions with NPs; it utilizes measurements performed in human cell culture systems *in vitro*, to develop a mechanistic mathematical model that can support analysis and prediction of *in vivo* effects of NPs. The model considers basic cellular mechanisms including proliferation, apoptosis, and production of cytokines in response to NPs. This new model is implemented for macrophages and parameterized using *in vitro* measurements of changes in cellular viability and mRNA levels of cytokines: TNF, IL-1b, IL-6, IL-8, and IL-10. The model includes *in vitro* cellular dosimetry due to nanoparticle transport and transformation. Furthermore, the model developed here optimizes the essential cellular parameters based on *in vitro* measurements, and provides a “stepping stone” for the development of more advanced *in vivo* models that will incorporate additional cellular and NP interactions.

## 1. Introduction

**Figure 1:**
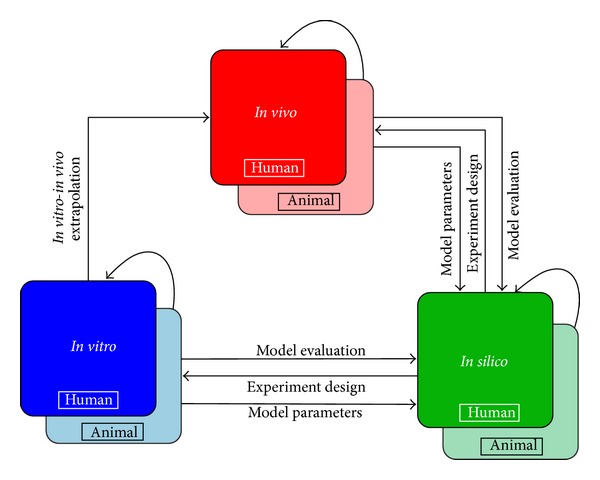
Schematic representation of information flow between complementary approaches,* in vitro*,* in vivo*, and* in silico*, in a framework aiming to support biological understanding of toxicodynamic processes in humans and model organisms.

*In vitro* testing of cellular responses to xenobiotics is an important alternative approach to animal experimentation in the context of human health risk assessment. This approach is supported by the 2007 National Research Council (NRC) report, “Toxicity testing in the 21st century: a vision and a strategy” [1], which called for a transformation of toxicity testing from a system based on whole animal testing to one focusing primarily on* in vitro* methods. This approach includes the use of selected* in vitro* assays in cell culture systems for hazard screening and development of quantitative structure activity models, limited animal studies for understanding kinetics, and the use of pharmacokinetic models for extrapolation of results from* in vitro* to* in vivo*, between species, and across sensitive populations [1]. The Tox21 initiative [2] forwarded jointly by the National Toxicology Program (NTP) and the Environmental Protection Agency (EPA) also recommends the increased use of* in vitro* assays for human toxicity assessment. Since the publication of the 2007 NRC report, there has been a considerable research effort on replacing experiments using animal* in vivo* models with a combination of targeted cellular* in vitro* experiments and computational modeling, to generate human risk estimates, thus saving effort and time and reducing uncertainty in cross-species scaling.  Figure 1 shows a simplified schematic representation of the complimentary relationship between* in vitro* and* in vivo* approaches for both animals and humans. The entire process involves multiple instances of information exchange between complementary strategies to lead to a complete understanding of human health risk assessment from environmental xenobiotics like NPs. The work presented here pertains to a model that aims to improve the understanding of toxic interactions occurring in an* in vitro* system, thus building an essential component of the framework of Figure 1, which can be used for the development of* in vivo* assessments in animals and in humans.

The two major steps involved in cellular interactions with xenobiotics are cellular uptake (which includes adhesion to the cell surface and internalization) and cellular immune responses due to the entry of the xenobiotic [3]. The immune response becomes critical during exposure to nanoparticles and might lead to cell apoptosis or altered cellular functions in response to secondary stimuli. The inflammatory response system in mammals consists of a series of cascading events facilitated by several types of cells and protein mediators such as cytokines and chemokines [4]. The major types of lung cells involved in the inflammatory response system are macrophages (Mph), dendritic cells, alveolar epithelial type I and type II (AT1 and AT2) cells, and the various inflammatory cells such as poly-mono-nuclear neutrophils (PMNs), lymphocytes, and eosinophils [5]. Xenobiotics can trigger cytokine and chemokine production when in the local milieu and once inside the cell. On release, these chemical mediators signal the influx of more macrophages and inflammatory cells into the lung from the blood circulation, inducing further a cascade of events which comprise an inflammatory response, leading to removal of the nanoparticles (NPs) due to phagocytosis and endocytosis by the inflammatory cells [6]. Such a response is expected to restore homeostasis after removal of the xenobiotic chemical and replenishment of the dead cells. Under normal circumstances, an inflammatory response is tightly controlled by release of both pro- and anti-inflammatory mediators [7]. However, in some cases, the response might be unable to revert to homeostasis, leading to tissue sepsis [7].

**Figure 2:**
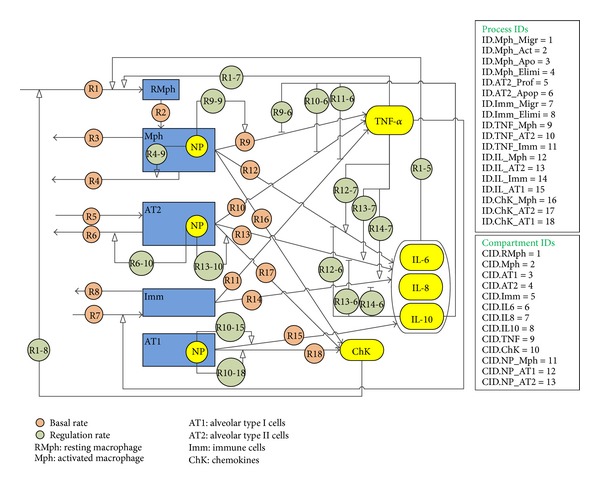
Cellular interaction, migration, and cytokine secretion of four cell types in the pulmonary alveolar subsystem for a proposed* in vivo* toxicodynamic model. The diagram includes macrophages, alveolar type I and alveolar type II cells, and immune cells, including cell-cell regulatory and signaling pathways. *R*1, *R*2, and so forth are the basal kinetic rates of the various cellular processes; *R*_*i*−*j*_ is the rate of regulation of process *R*_*i*_ by the cell or chemical in CID *j*. All process and compartment IDs are listed on the right. (The diagram follows the standards of the Systems Biology Graphical Notation (SBGN); see http://www.sbgn.org).

In the early stage of inflammation, elimination of the xenobiotic (e.g., NPs) by phagocytosis is the priority of the response system [8]. Macrophages (Mph) play a major role in the phagocytic removal of NPs, after which they migrate to the lymph glands through the lymphatic and blood circulation system or may be transferred to the throat via the mucociliary clearance system and swallowed or expectorated. NPs are also endocytosed by other cells of the alveolar region. Inside the cells, large quantities of reactive intermediates (reactive oxygen and nitrogen) are produced in the early stage to set up an appropriate response and neutralize the xenobiotics [9]. However, excess production of reactive intermediates also triggers secondary mechanisms which might lead to cellular apoptosis [6]. As mentioned earlier, the presence of NPs signals the influx of more phagocytic cells to the alveolar region for removal of NPs, partly involving release of cytokines and chemokines, which are produced by cells such as Mph, immune cells (Imm), comprising neutrophils, and lymphocytes in varying amounts [10]. As inflammation progresses, there is an increase in the cell count of the system due to influx of Mph and Imm and also an increase in concentration of the chemical mediators. Inflammatory chemical mediators can be proinflammatory or anti-inflammatory or both, depending on their concentration [11]. In essence, proinflammatory mediators (such as TNF-*α* and IL-6) upregulate and anti-inflammatory mediators (IL-10) downregulate the inflammatory response. In ideal cases, after the NPs have been removed from the system, the anti-inflammatory response is expected to help restore the system to homeostasis [11]. This is accompanied by removal of apoptotic cells, reduction in concentrations of proinflammatory cytokines, and clearance of immune cells from the tissue by migration or apoptosis [7].  Figure 2 summarizes the signaling effects mediated by the various alveolar cells in response to xenobiotic exposure.

Cellular dosimetry of nanoparticles in an* in vitro* cell culture medium is often ignored but is of crucial importance in estimating the actual number of particles reaching the cells in question [12, 13]. Medium properties affecting diffusion, sedimentation, agglomeration, and dissolution of the particles cause appreciable changes in particle distribution in the medium over time. Particle characteristics such as size, shape, surface coating, density, and agglomeration state and properties of the medium, such as density and viscosity, affect particokinetics in fluid media and need to be considered explicitly [14]. Aggregation of NPs in the culture medium causes change in size and shape of the NPs, which affects the cellular uptake of NPs. Dissolution of silver NPs leads to the production of ionic silver which is a known oxidizing and cytotoxic agent.

Mathematical modeling of cellular dynamics has been accomplished mechanistically utilizing systems of ordinary differential equations (ODEs) to simulate cellular processes [15, 16]. The model developed here combines the effects of NP transformation processes and cellular dynamics and has been implemented for macrophages comprising* in vitro* cultures. Mathematical modeling of cellular responses to xenobiotics requires estimation of a large number of parameters for the particular cell types under consideration.* In vitro* toxicological studies of the cellular response to xenobiotics allow for a simplified system that excludes confounding factors introduced by other cells and processes involved in tissue dosimetry. Assessments of risks due to NPs pose serious challenges to the NRC paradigm, particularly in the area of cellular dosimetry [17] and in the extrapolation to a real human population [2]. Derivation of parameters for such studies can be facilitated by modeling first the* in vitro* case, in conjunction with parameter optimization based on results of* in vitro* experiments. This information can provide a foundation for subsequent* in vivo* modeling of cellular responses in animal systems.

## 2. Methods

### 2.1. Modeling Transport and Transformation of Particles in Culture Media

Particle interactions need to be considered for* in vitro* systems to study toxic effects on biological media as these interactions impact particle dosimetry to the cells, affecting the actual number of particles that interact with cells at any given time [17]. Particle agglomeration is also an important process, especially for nanoparticles, whose large surface area to volume ratio leads to an increased tendency to agglomerate in order to reduce the overall surface energy of the system. In an actual* in vitro* medium, the processes of gravitational sedimentation, diffusion, agglomeration, and dissolution occur simultaneously. The first two processes are particle transport processes and the last two are particle transformation processes. A comprehensive model named ADSRM (agglomeration-diffusion-sedimentation-reaction model) has been developed for this purpose [18], to simultaneously quantify the* in vitro* evolution of NPs with time, considering agglomeration, sedimentation, and dissolution. The model has been implemented for citrate and PVP (polyvinylpyrrolidone) coated NPs to estimate* in vitro* dosimetry for the cell cultures. Nanoparticles denoted by Ag20 and Ag50 are citrate-stabilized silver nanoparticles produced according to Leo et al. [19]. The nanoparticles C20, P20, C110, and P110 were obtained from (and physicochemical properties were characterized by) the Nanotechnology Characterization Laboratory (NCL, National Cancer Institute at Frederick, SAIC-Frederick, Inc., Frederick, MD) under NIEHS-NCL Agreement. PVP of two different molecular weights were used as stabilizer, with 10 kD PVP present in 20 nm nAg and 40 kD PVP in 110 nm nAg. The properties of the nAg used for the study are summarized in Table 1. For* in vitro* cultures with cells, the nAg samples were diluted in RPMI1640 supplemented with 10% pooled human AB serum and sonicated in a Branson 3510 water bath sonicator for 2 minutes prior to addition to the cell cultures. The ADSRM uses the Direct Simulation Monte Carlo (DSMC) method to simulate evolution of a group of NPs in a medium and explicitly includes mutual collisions, diffusion, settling, and reactions of NPs with other chemicals in the medium. Reactions of coating chemicals like citrate and PVP have also been included in the model. Dissolution has been modeled as a surface-reaction controlled process and is affected by the exposed surface area of the nAg which changes due to oxidation of citrate [20] and dissolution of PVP [21]. Sulfidation of nAg has been shown to be a major process affecting silver precipitation in biological media [22]. Sulfidation might act as a potential detoxifying process by removing silver ions from solution [23, 24]. Liu et al. [24] have shown sulfidation to proceed via two mechanisms: direct, involving surface oxysulfidation of silver nanoparticles by sulfides, and indirect, involving precipitation of soluble silver ions as silver sulfides. Both processes of sulfidation have been included in the ADSRM to account for the presence of sulfides in cell culture media, using kinetic rates of sulfidation (both direct and indirect) from Liu et al. [24].

### 2.2. Mathematical Modeling of Inflammatory Response

#### 2.2.1. Modeling Equations

**Figure 3:**
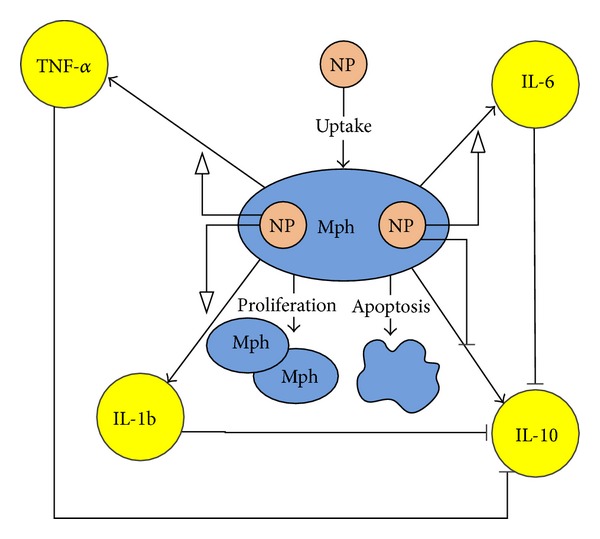
Schematic representation of macrophage dynamics involving proliferation, apoptosis, and cytokine secretion* in vitro*. (The diagram follows the standards of the Systems Biology Graphical Notation (SBGN); see http://www.sbgn.org).

Macrophages (Mph), alveolar type I (AT1) and alveolar type II cells (AT2), and inflammatory cells (Imm) are key components of the inflammatory response system in the alveolar region of the lung. The cellular compartments are responsible for the removal and intake of NPs and production of anti- or proinflammatory chemical mediators. In an* in vitro* system, the presence of only a single cell type allows for the analysis of the effects due to that specific type of cell, without interference of intercellular signaling effects.  Figure 3 shows the effects considered in the model for macrophages (Mph). The model considers macrophage proliferation and apoptosis and the production of four key cytokines in response to uptake of nAg from the culture media. For the* in vitro* model, the process is designed to start from the initial number of cells used in each sample medium. The cellular count is controlled by their apoptosis and proliferation. The proliferation of cells is assumed not to be limited by the presence of nutrients in the medium. These processes can be represented by the equations below:

(1) $$\begin{array}{c} \frac{dN}{dt}=R_{\text{Pro}}-R_{\text{Apo}}, \end{array}$$

where *R*_Pro_ is the rate of cellular proliferation and *R*_Apo_ is the rate of cellular apoptosis.

Cytokines TNF, IL-6, IL-1b, and IL-10 are considered to be secreted by the cell in the* in vitro* culture at a basal rate *R*_*i*_ and to degrade at a rate *k*_*d*,*i*_. The production of cytokines by cells is influenced by xenobiotics or cytokines present in the environment. The regulation is modeled using Hill-type kinetics. It is assumed here that the regulation is uniform over each cell type. Consider

(2) $$\begin{array}{c} \frac{dC_{i}}{dt}=R_{i}\left(1+f_{\text{reg},i}\right)-k_{d,i}C_{i},\\ f_{\text{reg},i}=\underset{i,j}{\prod }f_{i-j}, \end{array}$$

where *f*_*i*−*j*_ denotes the regulation effect of cytokine *j* on cytokine *i*. The regulation effects are modeled via Hill-type equations as follows:

(3) $$\begin{array}{c} f_{i-j}=\frac{C_{j}^{n}}{x_{i-j}+C_{j}^{n}},\;\text{for}\;\;\text{upregulation}\\ f_{i-j}=\frac{x_{i-j}}{x_{i-j}+C_{j}},\;\text{for}\;\;\text{downregulation}. \end{array}$$

The power *n* controls the strength of the regulation effect.

#### 2.2.2. Cellular Uptake of Nanoparticles

NPs are taken up by alveolar cells via endocytosis or phagocytosis. This phenomenon plays a critical role in estimating exposure and fate of NPs in the biological system as the alveolar epithelial cells form the gateway to the circulatory system and hence to the entire body. Lai et al. [25] showed that charcoal NPs are significantly taken up by type I cells, type II cells, and macrophages. Cellular uptake of particles is influenced by particle type, size, and surface charge [26]. Cellular uptake has been considered to be composed of two processes: delivery and adhesion of NPs onto the cell and uptake of NPs by the cell via phagocytosis. Adhesion of NPs onto the cell surface is a function of particle size, surface zeta potential, and cell type. Adhesion probability, *k*_*f*,*m*_, is modeled according to Su et al. [26] as *k*_*f*,*m*_ = *k*_*c*,*m*_ *η*_*o*_ *η*_*e*_((1 − *ϵ*)/*ϵd*_*c*_), where *ϵ* is the tissue porosity for Mph, *d*_*c*_ is the cell diameter, *k*_*c*_ is a cell type dependent parameter, and *η*_*o*_, *η*_*e*_ are the relative affinities of particle adhesion to the cell due to their size and surface zeta potential, respectively. Values of porosity and average cell diameter for Mph have been obtained from Clegg et al. [27] and Morgan and Talbot [28]. *η*_*o*_ is a function of NP diameter *d*_*p*_; the relation has been obtained for alveolar Mph from Oberdörster et al. [29]. *η*_*e*_ is a function of *ζ*, the surface zeta potential of the NPs; the relation was obtained for alveolar Mph from Tabata and Ikada [30]. Mph phagocytosis has been modeled by Michaelis-Menten kinetics, with phagocytosis rate parameters estimated from Beduneau et al. [31]. The uptake of NPs by cells is given by

(4) $$\begin{array}{c} \frac{dN_{\text{NP}}}{dt}=-R_{\text{NP},\text{Mph}}, \end{array}$$

where *R*_Mph_ = *k*_*f*,*m*_(*V*_*m*_ *N*/(*K*_*m*_ + *N*)) and *k*_*f*,*m*_ = *k*_*c*,*m*_((1 − *ϵ*)/*ϵd*_*c*_)*η*_*o*_ *η*_*e*_, where *η*_*o*_ = *f*(*d*_*p*_), *η*_*e*_ = *f*(*ζ*).

### 2.3. Cell Culture Measurements

Measurements of cell viability were made with Cell Titer 96 Aqueous One Solution Cell Proliferation Assay [MTS, (3-(4,5-dimethylthiazol-2-yl)-5-(3-carboxymethoxyphenyl)-2-(4-sulfophenyl)-2H-tetrazolium)] with human alveolar macrophages as well as with human monocyte-derived macrophages (MDMs), incubated with different doses of 20 nm and 110 nm nAg for 24 hours. The alveolar macrophages are primary human cells, cultured and exposed to nAg in serum free DCCM-1 media, with approximately 100,000 cells/well. The experiment was carried out in 96-well plates in a volume of 200 *μ*L. The human MDMs were incubated with nAg in RPMI1640 medium (supplemented with L-glutamine and 10% pooled human AB serum) in a total volume of 1.5 mL. The cytokine study was carried out with human MDMs. NP solutions were prepared as 2, 20, and 50 *μ*g/mL and sonicated in a Branson 3510 water bath sonicator for 2 minutes prior to* in vitro* cell exposure. 0.75 mL of the media was added to the cells making the total volume 1.5 mL. This resulted in an actual NP dose of 1.5, 15, and 37.5 *μ*g to the cell medium containing about 44,000 cells per well. Total RNA extracted from exposed MDMs was analyzed by quantitative RT-PCR as described by Sarkar et al. [32].

## 3. Results and Discussions

### 3.1. ADSRM Simulation

**Figure 4:**
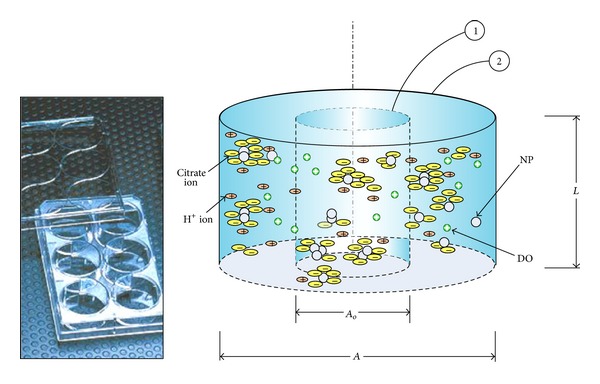
Schematic showing the implementation of the ADSRM for citrate-stabilized nAg in an* in vitro* culture well, with the control volume and the actual well shown as 1 and 2, respectively, along with an actual image (inset) of a Falcon 6-well plate used for* in vitro* measurements (figure reproduced from Mukherjee et al. [18] with permission) (diagram is not drawn to scale and is representative only).

**Figure 5:**
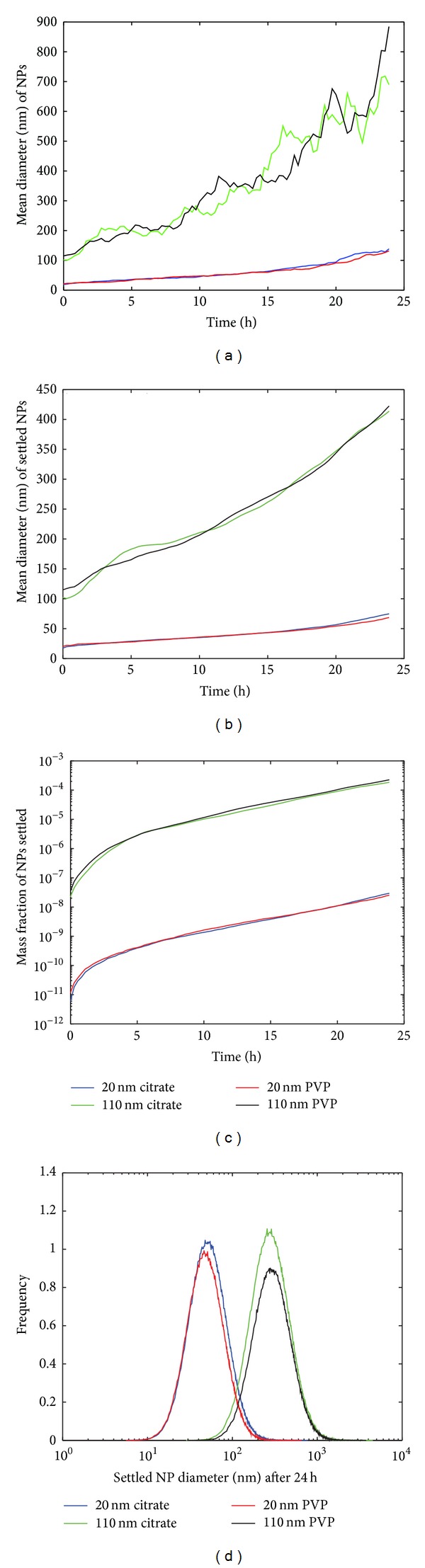
Model predictions from ADSRM for transformation processes of 4 different types of nAg in cell culture media over 24 hours; (a) mean diameter of all NPs in the medium over the time span of 24 hours, (b) mean diameter of NPs settled at the bottom of the culture plate, (c) mass fraction of nAg settled, and (d) size distribution of nAg settled. ((a) and (c) reproduced from Mukherjee et al. [18] with permission).

The ADSRM was run for a time period of 24 hours, using the NP properties summarized in Table 1 and the parameter values summarized in Table 2. The ADSRM model was implemented for a cell culture plate simulating a Falcon 6-well plate, as shown in Figure 4.  Figure 5 shows the changes in nAg mean diameter and size distribution over 24 hours. In the* in vitro* culture, the cells reside at the bottom of the culture plate and consequently are not exposed to the entire population of NPs in the medium. Only the fraction of NPs which have settled (Figure 5(c)) interact with the cells. The figures show that the average NP diameters increase due to agglomeration. Between 20 nm and 110 nm nAg, the larger nAg have a greater preference toward settling, leading to a relatively higher dose for the cells at the bottom of the culture plate.

### 3.2. *In Vitro* Cell Model Results

**Figure 6:**
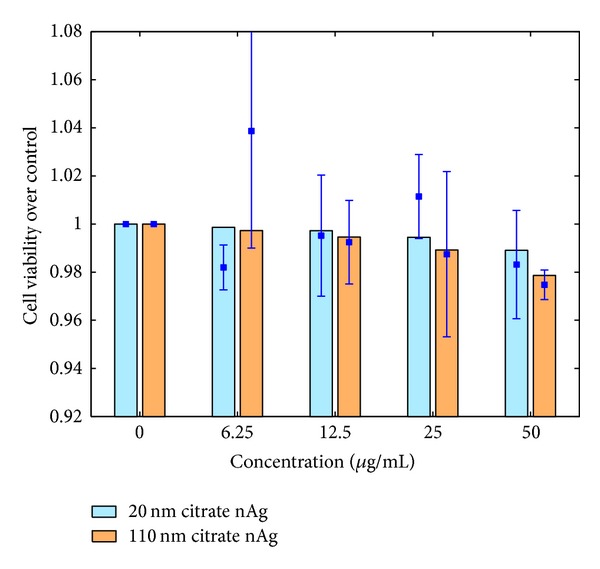
Comparison between model prediction and* in vitro* measurement for human alveolar macrophage cell viability, 24 hours after incubation. Bars represent model predictions and squares and error bars represent* in vitro* measurements.

**Figure 7:**
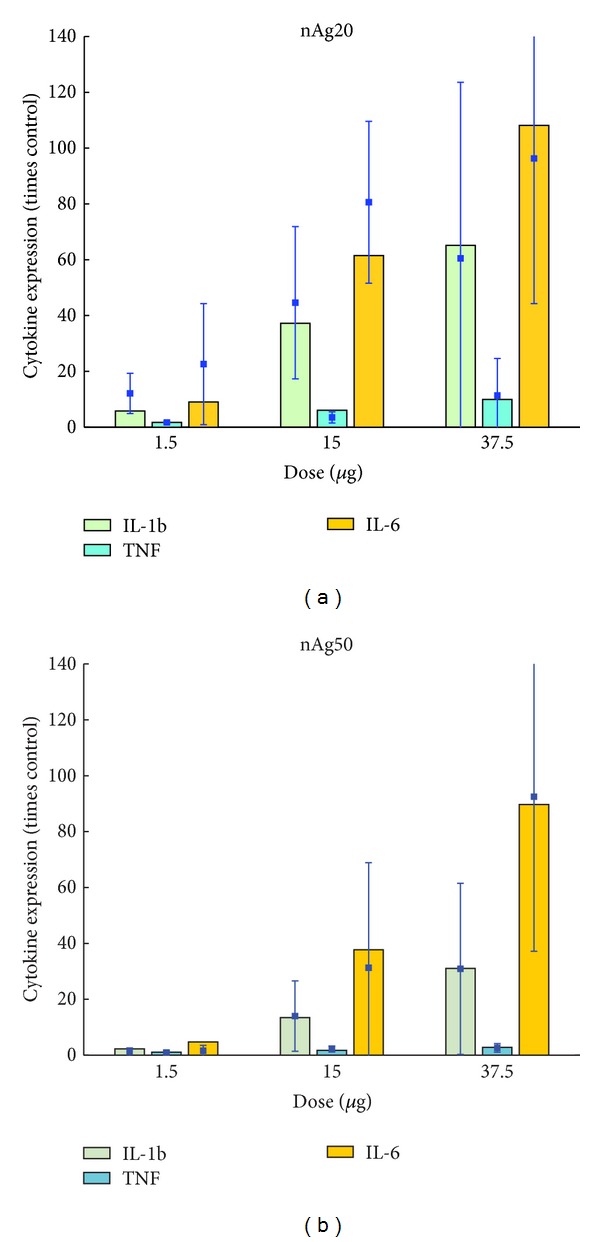
Comparison of model predictions and measured values of proinflammatory cytokine levels in culture medium after 4 hours for human MDMs with different doses of 20 nm (a) and 50 nm (b) nAg* in vitro*. Bars represent model predictions and squares and error bars represent* in vitro* measurements.

**Figure 8:**
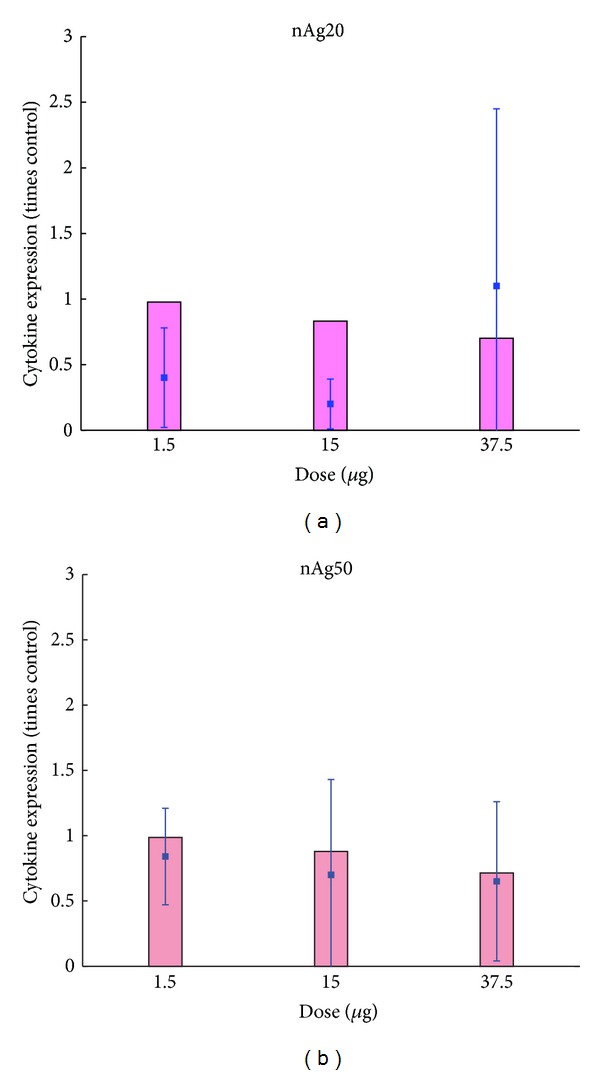
Comparison of model predictions and measured values of anti-inflammatory cytokine (IL-10) levels in culture medium after 4 hours for human MDMs with different doses of 20 nm (a) and 50** **nm (b) nAg* in vitro*. Bars represent model predictions and squares and error bars represent* in vitro* measurements.

**Figure 9:**
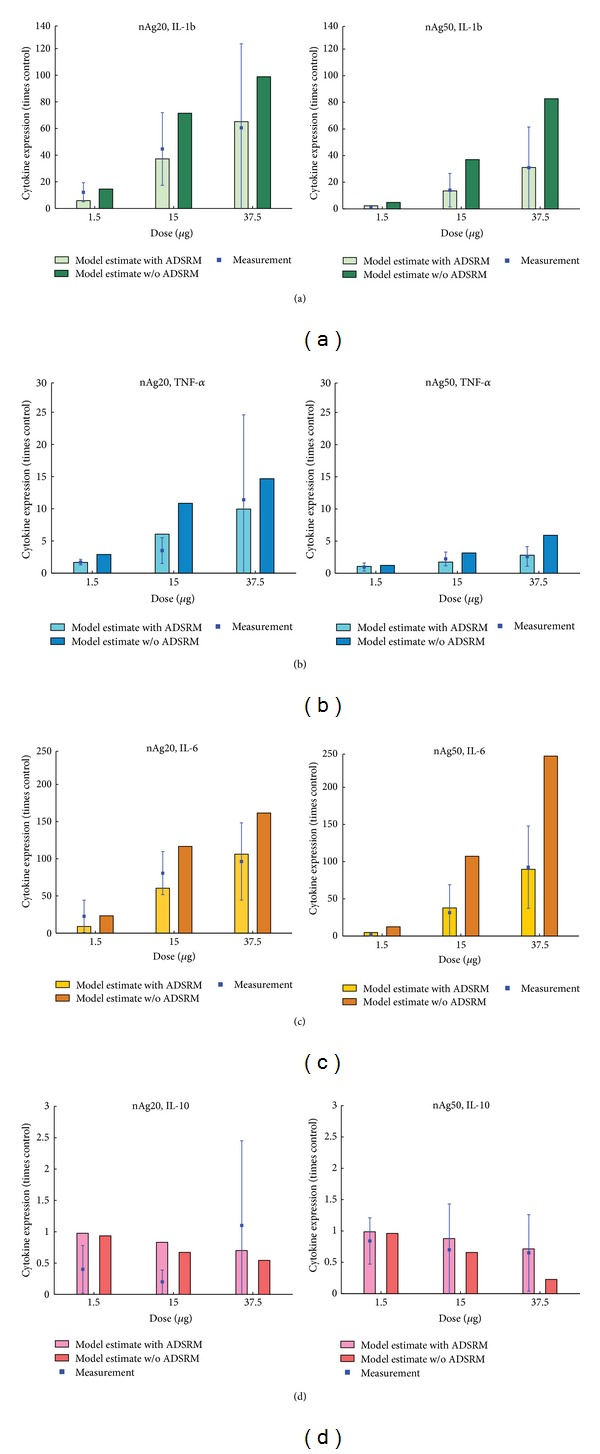
Comparison of model predictions with and without using ADSRM for cellular dosimetry estimation against measured values of cytokine levels in culture medium with different doses of 20 nm and 50 nm nAg* in vitro* with 4-hour incubation.

Results from the mathematical model of the* in vitro* cell system described above are presented and compared with* in vitro* measured values performed for this purpose. The parameter values have been summarized in Table 3. Cell viability was measured* in vitro* for human alveolar macrophages using MTS assays.  Figure 6 shows comparisons between model predictions and measured values for four doses of 20 and 110 nm citrate-coated nAg. There is good agreement between model predictions and measured values, except for the dose of 6.25 *μ*g/mL of 110 nm nAg for which the measurement shows an unusually high value but also a correspondingly high error.  Figure 7 shows a comparison between model predictions and measured values for proinflammatory cytokine (IL-1b, TNF-*α*, and IL-6) levels in the cell culture medium. Cytokine mRNA levels were measured in cell culture medium with human MDMs 4 hours after incubation with 20 and 50 nm citrate-coated nAg. Model predictions and measured values seem to agree well for IL-1b and TNF-*α*; however the model seems to consistently underestimate the level of IL-6 cytokine.  Figure 8 shows the same comparison for the anti-inflammatory cytokine IL-10. Model predictions agree well with measured values, except for the highest dose of 20 nm nAg, where the measured value shows a high degree of uncertainty. Additionally, the model was executed with and without the inclusion of the NP agglomeration-diffusion-sedimentation-reaction model (ADSRM), to determine the extent of effects due to* in vitro* cellular dosimetry of NPs.  Figure 9 shows a comparison between model predictions with and without ADSRM-based adjustments along with a comparison of measured values for the four different cytokines modeled. The results show a considerable increase in the levels of proinflammatory cytokines and a decrease in the anti-inflammatory cytokines when ADSRM is not considered. Absence of cellular dosimetry calculations presumes a well-mixed culture medium, where all the particles come into contact with the cells instantly, giving a proportionately higher effect than what is observed in the measured values.

### 3.3. Discussion

The model was parameterized to obtain values of rate constants for cell proliferation, apoptosis, and cytokine secretion. The effect of NPs on cellular processes was also parametrized. The parameters optimized in this study can be used to support predictions in the context of* in vivo* cellular inflammatory modeling which will consider various cell types in physiological interactions and simulate the actual alveolar microenvironment.* In vivo* modeling of NP toxicity can be informed by the cellular parameters estimated from this work but of course needs to include other cellular interaction and signaling effects. Inflammatory processes* in vivo* are composed of multiple cellular signaling effects which regulate cytokine secretion and cellular migration in a tissue [4].  Figure 2 presents a diagram summarizing the key signaling and regulatory processes occuring in the alveolar subsystem involving type I cells, type II cells, macrophages, and immune cells. Immune cells like lymphocytes and neutrophils are recruited from the blood stream at sites of inflammation. Macrophage dynamics are also affected by complexities which were not considered in this* in vitro* model. Macrophages are known to present markedly different phenotypes in tissue systems, a fraction of them being in a “resting” phase and others in an “active” phase. They also demonstrate well known M1 and M2 phenotypes [11] wherein they support proinflammatory and anti-inflammatory actions, respectively. Various basal kinetic rates and intercellular regulatory rates are depicted by circles in Figure 2.  Table 4 summarizes a mathematical framework for implementation of the inflammatory pathway model* in vivo* using parameter values and information from* in vitro* models as described here. Macrophage and immune cell recruitment, macrophage activation, cytokine secretion, and various intercellular activation and inhibition processes would be caused by NP exposure* in vivo* and these processes will be modeled using differential equations shown in Table 4. These processes would also be affected by parameters such as *k*_*f*_ and *k*_*c*_ which in turn are functions of NP and cell properties as has been discussed in detail in Section 2.2.2. The regulation processes are expected to follow the basic scheme shown for macrophages in Equations (2) and (3) but would be more complex, involving additional regulatory parameters.* In vivo* modeling of cellular dynamics would also require a complete whole body toxicokinetic model to predict NP translocation and retention utilizing* in vivo* data in rodents [33–35]. Particularly, the rate *R*_NP,alv_ shown in Table 4 would capture the rate of translocation of NPs into the alveolar region after inhalation.  Figure 1 shows a simplified representation of a framework utilizing information from various* in vitro* and* in vivo* studies in conjunction with* in silico* models like the one described in this article, to support better understanding of nanoparticle toxicodynamics. A combination of* in vitro* and* in vivo* models along with suitable* in vitro-in vivo* extrapolation can help utilize information from high throughput* in vitro* toxicological studies and incorporate such information in building detailed predictive models [36] offering novel insights into complex biological processes.

## Figures and Tables

**Table 1:** Properties of silver NPs used in the model.

NP	Coating	Core material	Density (g/cm^3^)	Mol. wt.	Coating mol. wt	Zeta potential (mV)
Ag20	Citrate	Ag	10.49	108	258	−39.2
Ag50	Citrate	Ag	10.49	108	258	−39.2
C20	Citrate	Au	10.87	115.3	258	−44.3
P20	PVP	Au	10.87	115.3	10000	−38.2
C110	Citrate	Au	10.49	108.04	258	−45.2
P110	PVP	Au	10.49	108.04	40000	−31.6

Source: nAg properties (for Ag20, Ag50) from Leo et al., 2013 [19]; nAg properties (for C20, P20, C110, and P110) from http://www.nanoComposix.com.

**Table 2:** Parameter values used in the ADSRM implementation.

Parameter	Value	References∖notes
Packing factor	0.637	Sterling et al. [37]
Fractal dimension	2.3	Hinderliter et al. [17]
Activation energy	33 × 10^3^ J	Zheludkevich et al. [38]
Rate constant for citrate oxidation	1.235 × 10^−10^ mol/m^3^/sec	Estimated from Zhang et al. 2011 [20]
Rate constant for direct sulfidation	0.018 mM^−1^ *·*min^−1^	Liu et al. 2011 [24]
Rate constant for indirect sulfidation	0.00016 min^−1^	Liu et al. 2011 [24]
Mass transfer rate of O_2_	1.67 × 10^−6^ mol/m^3^/sec	Estimated by Zhang et al. 2011 [20]
Saturation conc. of O_2_	8.96 mg/L	Zhang et al. 2011 [20]
Density of medium	1000 kg/m^3^	Density of water
Viscosity of medium	0.001 Pa-s	Viscosity of water

**Table 3:** Optimized values of parameters for MDM *in vitro* cultures (with reference for initial estimate).

Proliferative index	0.23	[39]
Apoptotic index	0.11	[39]
TNF production rate∗	2.671 × 10^−10^ nmol/min	[10]
IL-6 production rate∗	7.0962 × 10^−10^ nmol/min	[10]
IL-8 production rate∗	4.34 × 10^−8^ nmol/min	[10]
IL-10 production rate∗	9.458 × 10^−10^ nmol/min	[40]

*Cytokine production rates represent the rate for 10^6^ cells.

**Table 4:** Equations constituting the mathematical framework for a proposed *in vivo* cellular scale inflammatory pathway model.

Resting macrophages	$\frac{dN_{\text{RMph}}}{dt}=R_{\text{Mig,RMph}}-R_{\text{Act}}$
Active macrophages	$\frac{dN_{\text{Mph}}}{dt}=R_{\text{Act}}-R_{\text{Elim,Mph}}-R_{\text{Apo,Mph}}$
Type I cells	$\frac{dN_{\text{AT1}}}{dt}=R_{\text{Pro,AT1}}-R_{\text{Apo,AT1}}$
Type II cells	$\frac{dN_{\text{AT2}}}{dt}=R_{\text{Pro,AT2}}-R_{\text{Apo,AT2}}$
Immune cells	$\frac{dN_{\text{Imm}}}{dt}=R_{\text{Mig,Imm}}-R_{\text{Elim,Imm}}$
NP uptake by Mph	$\frac{dN_{\text{NP,Mph}}}{dt}=k_{\text{f,Mph}}\cdot \frac{V_{\text{Mph}}N_{\text{NP}}}{K_{\text{Mph}}+N_{\text{NP}}}$
NP uptake by AT1	$\frac{dN_{\text{NP,AT1}}}{dt}=k_{\text{f,AT1}}\cdot \frac{V_{\text{AT1}}N_{\text{NP}}}{K_{\text{AT1}}+N_{\text{NP}}}$
NP uptake by AT2	$\frac{dN_{\text{NP,AT2}}}{dt}=k_{\text{f,AT2}}\cdot \frac{V_{\text{AT2}}N_{\text{NP}}}{K_{\text{AT2}}+N_{\text{NP}}}$
TNF-α secretion	$\frac{dM_{\text{TNF}}}{dt}=R_{\text{TNF,Mph}}+R_{\text{TNF,AT2}}+R_{\text{TNF,Imm}}$
IL-6 secretion	$\frac{dM_{\text{IL6}}}{dt}=R_{\text{IL6,Mph}}+R_{\text{IL6,AT1}}+R_{\text{IL6,AT2}}+R_{\text{IL6,Imm}}$
IL-8 secretion	$\frac{dM_{\text{IL8}}}{dt}=R_{\text{IL8,Mph}}+R_{\text{IL8,AT1}}+R_{\text{IL8,AT2}}+R_{\text{IL8,Imm}}$
IL-10 secretion	$\frac{dM_{\text{IL10}}}{dt}=R_{\text{IL10,Mph}}+R_{\text{IL10,AT1}}+R_{\text{IL10,AT2}}+R_{\text{IL10,Imm}}$
Chemokine secretion	$\frac{dM_{\text{ChK}}}{dt}=R_{\text{ChK,Mph}}+R_{\text{ChK,AT1}}+R_{\text{ChK,AT2}}$
NP balance	$\frac{dN_{\text{NP}}}{dt}=R_{\text{NP,alv}}-R_{\text{NP,Mph}}-R_{\text{NP,AT1}}-R_{\text{NP,AT2}}-R_{\text{Elim}}$
